# Discovery of biosynthetic enzymes for β-D-*manno*-heptoses across kingdoms: novel agonists for ALPK1/NF-κB-dependent immune response

**DOI:** 10.1038/s41392-024-02003-y

**Published:** 2024-10-08

**Authors:** Gunter Maubach, Michelle C. C. Lim, Michael Naumann

**Affiliations:** Otto von Guericke University, Institute of Experimental Internal Medicine, Medical Faculty, Magdeburg, Germany

**Keywords:** Infection, Molecular medicine, Microbiology

**Fig. 1:**
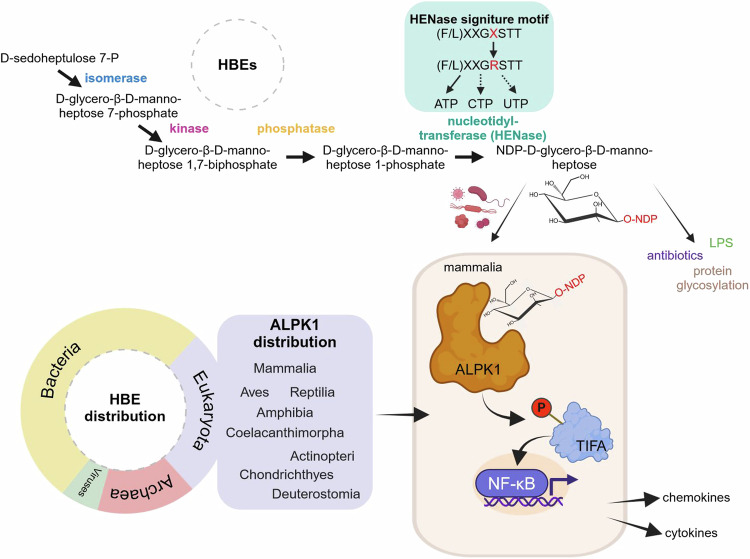
New findings on NDP-heptoses as agonists for the immune response. Small molecule metabolites such as ADP-heptose are synthesized by HBEs exhibiting isomerase, kinase, phosphatase, and nucleotidyltransferase activities. Of note, three subgroups of HBEs with nucleotidyltransferase activity (HENases) exist, exhibiting solely this activity or combined with kinase, or isomerase/kinase activities. In bacteria, where HBEs were first reported, they catalyzed the four-step biosynthesis of ADP-heptose starting from D-sedoheptulose 7-phosphate. Functional HBEs are prevalent in bacteria, archaea, viruses, and some eukaryotes. The authors discovered the presence of a widely conserved arginine residue at the fifth N-terminal position of the (F/L)XXGXSTT motif (STT_R5_) in HENases that enable them to synthesize also CDP- and UDP-heptoses. A striking feature of the NDP-heptoses is their ability to act as immunostimulants. Pathogenic organisms deliver NDP-heptoses into mammalian cells, where they are detected by ALPK1, triggering its kinase activity. The ensuing TIFA phosphorylation initiates a signaling cascade to activate NF-κB, leading to the release of cytokines and chemokines that result in the recruitment of immune cells. In addition, NDP-heptoses could also serve as building blocks for protein glycosylation, or the production of LPS or antibiotics. The figure is created with BioRender.com

A recent study by Tang et al. ^1^ in *Science* reveals the cross-kingdom widespread occurrence of functional nucleotide-diphosphate (NDP)-heptose biosynthetic enzymes (HBEs) that accounts for the synthesis of NDP-heptoses to activate the alpha-protein kinase 1 (ALPK1)-dependent innate immune response. This study not only highlights the importance of the metabolite β-D-*manno*-heptose as pathogen-associated molecular patterns (PAMPs) but also raises the question of possibly other biological roles, especially in the different kingdoms (Fig. 1).

The hypothesis of how innate immunity distinguishes ‘self’ from ‘nonself’ was first introduced in 1989,^2^ the central idea being establishing a link between innate immune signaling and activation of the adaptive immune response. Certain distinct and conserved components of pathogenic microorganisms, termed PAMPs, induce the innate immune response that results in the production of inflammatory mediators, e.g., chemokines and cytokines, to activate T cells, ensuring that adaptive immunity is triggered only in the presence of a threat. Three criteria are characteristic for PAMPs: (i) they have a common core structure among a broad range of microorganisms, (ii) they are products of pathways unique to microorganisms and (iii) they are essential for the survival or function of the microorganism.^2^ PAMPs can be divided into lipids, proteins, carbohydrates, and nucleic acids. In order to initiate the innate immune response, the host possesses an array of different germline-encoded molecules termed pattern recognition receptors (PRRs),^3^ e.g., Toll-like receptors, cyclic GMP-AMP synthase (cGAS) and retinoic acid-inducible gene-I (RIG-I)-like receptors, detecting extra- and intracellular PAMPs. PRRs consist of ligand-binding domains as well as signaling domains and require adapter molecules to transduce the signal across a signaling cascade. Interestingly, some PRRs have catalytic activities, for instance RIG-I and cGAS have an ATPase activity and a nucleotidyltransferase activity, respectively. ALPK1 is a recently discovered cytosolic PRR that exhibits uniquely a kinase activity.^4^ Binding of the PAMP ADP-heptose to the PRR ALPK1 activates ALPK1’s kinase activity to phosphorylate the threonine residue at position 9 of tumor necrosis factor (TNF) receptor-associated factor (TRAF)-interacting protein with a forkhead-associated domain (TIFA). Subsequent recruitment of other factors into so-called TIFAsomes culminates in the activation of classical and alternative nuclear factor ‘kappa-light-chain-enhancer’ of activated B-cells (NF-κB) pathways.^5^ Through evolutionary analysis, Tang et al. ^1^ showed that ALPK1 appears in some vertebrates, including mammals, after deuterostomes lost the ability to synthesize NDP-heptoses, indicating that ALPK1 evolved as a specific receptor for the immunological recognition of β-D-*manno*-heptose.

The multi-step biosynthesis of NDP-heptose starting from D-sedoheptulose 7-phosphate is catalyzed by different HBEs exhibiting isomerase, kinase, phosphatase, and nucleotidyltransferase activities. Notably, three subgroups of HBEs with nucleotidyltransferase activity (HENases) exist, exhibiting solely this activity or combined with kinase, or isomerase/kinase activities. Tang et al. ^1^ examined the ability to synthesize ADP-heptose across different kingdoms by first performing a homology search for HENases and then generating an intersection with the remaining three types of HBEs. Apart from the phosphatase being absent in viruses and eukaryotes, the authors extended the knowledge about the repertoire of functional HBEs beyond bacteria to archaea, viruses, and eukaryotes. Enzymatic assays using combinations of HBEs from the same organism (at least one from each kingdom was chosen) showed that they could all synthesize ADP-heptose. Importantly, the authors established convincingly through mutagenesis experiments that the presence of an arginine residue at the fifth N-terminal position of the consensus motif (F/L)XXGXSTT (STT_R5_) in HENases endows them with NTP substrate promiscuity, so that CDP- or UDP-heptose, or both could be additionally synthesized. The arginine residue seems to stabilize the interaction with the substrate by stacking to the nucleotide bases through cation-π interaction and forming hydrogen bonds with the phosphate groups. In addition, they found STT_R5_-containing HENases in all three subgroups and across the kingdoms of eukaryotes, archaea, and bacteria. However, STT_R5_ is not strictly necessary for NTP substrate promiscuity as seen for HldE_ST_ from *Salmonella typhimurium* SL3144. Although all three activated heptoses could activate the kinase activity of purified ALPK1 to phosphorylate TIFA in a dose-dependent manner with similar efficiencies, cell culture studies showed that CDP- and UDP-heptoses induced stronger TIFA phosphorylation and subsequent NF-κB activation. Consistently, injection of CDP- and UDP-heptoses into mice elicited higher secretion levels of NF-κB-driven inflammatory mediators compared to ADP-heptose. On the contrary, electroporation of ADP-, CDP- or UDP-heptose into 293 T cells with genome integration of *EGFP-TIFA* induced similar levels of TIFA phosphorylation and NF-κB activation. These observations collectively hint at possible differences in the delivery mechanism of the hydrophilic NDP-heptoses to host cells. This is of relevance particularly in the context of non-invasive pathogenic microorganisms. Invasive bacteria like *Neisseria* and *Shigella* release NDP-heptoses into the cytosol. In the case of non-invasive bacteria, delivery of sugar metabolites has been shown to be conjugation system-dependent, although endocytosis has also been implicated.^5^ Whether these two events are interconnected could be explored in future studies. On the same note, the delivery mechanism of NDP-heptoses from archaea to host cells represents a novel research area. How viruses are able to use the host translational system to produce ADP-heptose is still unclear.

Using *Burkholderia multivorans* and *Burkholderia cepacia*, two closely related species infecting respiratory organs, in in vitro cellular mimic assays, the authors demonstrated that it is possible for HBE-containing organisms to synthesize significant amount of NDP-heptoses and store them without toxic effects. Despite this, the open question remains if the concentrations of these stored activated heptoses are physiologically relevant. On the other hand, the utilization of NDP-heptoses by non-pathogenic organisms is eventually not related to NF-κB activation but serves as precursor for other processes, e.g., protein glycosylation in *Escherichia coli* via the *aah* (autotransporter-adhesin-heptosyltransferase) gene and synthesis of the antibiotic septacidin in *Streptomyces fimbriatus*.

By extrapolating the distribution of HBEs and the corresponding generation of NDP-heptoses beyond bacteria to include archaea, viruses, and eukaryotes, this study raises new questions about the biological roles of these metabolites. Could these metabolites act as PAMPs for the newly discovered kingdoms too or are they building blocks for complex biomolecules? Given the diversity of kingdoms, there are perhaps also yet undiscovered synthetic pathways to produce other small molecule metabolites.
